# Engineering of Metal–Organic Framework-Derived CoTiO_3_ Micro-Prisms for Lithium-Ion Batteries

**DOI:** 10.3390/molecules30010034

**Published:** 2024-12-26

**Authors:** Tao Li, Minghui Song, Qi Zhang, Yifan Li, Gengchen Yu, Xue Bai

**Affiliations:** 1 Department of Materials Engineering, Xuzhou College of Industrial Technology, Xuzhou 221140, China; 2 School of Materials Science and Engineering, Shandong University of Science and Technology, Qingdao 266590, China; 3 School of Environmental and Municipal Engineering, Qingdao University of Technology, Qingdao 266033, China

**Keywords:** metal–organic framework, lithium-ion batteries, cobalt titanate, anode materials, Ni doping

## Abstract

Metal–organic framework (MOF)-derived transition metal compounds and their composites have attracted great interest for applications in energy conversion and storage. In this work, hexagonal micro-prisms of Ni-doped CoTiO_3_ composited with amorphous carbon (Ni_x_CTO/C) were synthesized using Ti-Co-based MOFs as precursors. The experimental results indicate the substitutional doping of Ni^2+^ for Co^2+^ in CoTiO_3_ (CTO), leading to improved conductivity, as further confirmed by density functional theory calculations. Thus, the carbon-free sample of Ni-doped CTO exhibits improved lithium storage properties compared to the pristine one. Furthermore, when coupled with in situ-formed carbon, the dually modified Ni_0.05_CTO/C micro-prisms demonstrated a significantly increased reversible capacity of 584.8 mA h g^−1^, excellent rate capability, and superior cycling stability at a high current density of 500 mA g^−1^. This enhanced electrochemical performance can be attributed to the synergistic effect of Ni doping and carbon coating.

## 1. Introduction

With the increasing share of new clean energy in modern energy systems, various energy storage systems with a high capacity and long lifespan have developed rapidly in recent years. Lithium-ion batteries (LIBs), as one of the most common energy storage devices, have been widely used in many fields ranging from large-scale energy storage stations and electric vehicles to small electronic devices [1,2,3]. It is noteworthy that the demands of anode materials with high energy/power density, low cost, design flexibility, and long life cycles have become more urgent due to the considerable drawbacks of the relatively low theoretical capacity (372 mA h g^−1^) and poor rate capability of commercial graphite [4]. Furthermore, the low working potential of graphite anodes at around 0.1 V vs. Li/Li^+^ tends to induce the generation of Li dendrites, causing serious safety risks [5]. Though great progress has been made in improving the performance of graphite anodes, the search for alternative anodes with higher performance is still a challenge.

Transition metal oxides (TMOs) are capable of serving as one of the more striking anodes for LIBs due to their high theoretical capacities, which are two to three times higher than that of graphite [6]. For example, Co_3_O_4_ can deliver a high specific capacity of up to 890 mA h g^−1^ in theory, based on a multi-electron transfer reaction. However, conversion-reaction-based TMO materials usually suffer from rapid capacity fading due to the huge volume change during repeated lithium insertion/extraction processes. Among the various TMOs, titanium dioxide (TiO_2_), a typical intercalation-type anode material, has also received much attention owing to its high safety, low cost, and excellent stability, which results from the small volume change (<4%) during its cycle [7]. Despite the above-mentioned merits of TMOs, their inherited disadvantages, including their low electronic conductivity and poor cyclic properties, limit their practical applications in the next generation of high-energy LIBs. Aiming to enhance the electrochemical performance of TMOs, several effective modification strategies have been developed, including the following: (i) compositing and/or surface coating with conductive materials such as carbon materials to enhance electronic conductivity and to mitigate the effects of volume changes and aggregation of active nanoparticles on electrochemical cycling [8,9]; (ii) processing TMO materials to nanoscale size or porous nano- or microscale structures to increase the specific surface area and reactivity towards lithium ions as well as to improve the cycling stability [10]; and (iii) doping with heterogeneous elements (e.g., N, Co, Nb, etc.) to improve the electronic conductivity of TMOs [11,12,13]. In addition, grain size is also reduced after doping modification, which is conducive to the diffusion of lithium ions. However, most of the previous studies adopted single modification measures, while investigations of multiple modification strategies are rarely reported.

Bimetallic titanates (MTiO_3_, M = Mn, Co, Ni, etc.) have seen substantial applications in LIBs in recent years [14,15,16,17]. As a type of titanium containing bimetallic TMO, MTiO_3_ integrates the advantages of two suitable types of metal oxide. In particular, during the charge and discharge process, the mutual segregation of heterogeneous oxides can form a self-supporting network to alleviate the volume change [14]. Thus, MTiO_3_ can exhibit enhanced electrochemical performance compared with the corresponding single-metallic TMOs. Recently, the use of metal–organic frameworks (MOFs) as precursors to prepare transition metal compounds and their composites for energy storage and other applications has been demonstrated to be a simple and efficient method due to their unique structural characteristics and diversity [18,19]. The organic ligands contained in the precursors can be carbonized to amorphous carbon in situ through pyrolysis under specific atmospheres (e.g., Ar, N_2_), which effectively improves the stability and electrical conductivity of the MOF-derived composites. For example, Liu et al. prepared CoTiO_3_/C hexagonal micro-prisms (about 3 μm in size) using a cobalt–titanium–ethylene glycol bimetal–organic framework synthesized by the sol–gel method as a self-template [14]. Benefiting from a stable structure and MOF-derived carbon, the CoTiO_3_/C composite exhibited a charge capacity of 231.2 mA h g^−1^ at the 15th cycle at 500 mA g^−1^ and showed excellent cycling stability for 200 cycles.

Herein, we propose a simple dual-modification strategy of Ni doping combined with carbon coating to engineer CoTiO_3_ materials based on the MOF derivative method. The results show that a composite of Ni-doped CoTiO_3_/amorphous carbon (Ni_x_CTO/C) hexagonal micro-prisms was successfully synthesized and that Ni doping did not destroy the unique 1D structure. Furthermore, the optimized sample of Ni_0.05_CTO/C exhibited greatly enhanced electrochemical performance due to the synergistic effect, highlighting its potential as an anode material for LIBs.

## 2. Results and Discussion

**Figure 1:**
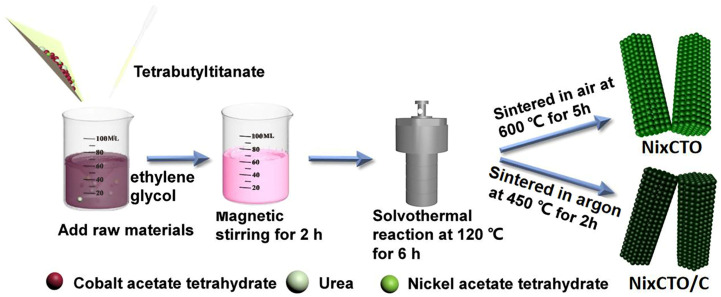
Schematic illustration of the synthesis procedure of Ni_x_CTO and Ni_x_CTO/C micro-prisms.

Figure 1 simply illustrates the preparation route of Ni_x_CTO and Ni_x_CTO/C micro-prisms. Specifically, ethylene glycol served as both the solvent and complexing agent [20]. With the addition of raw materials, the mixed solution gradually changed from a transparent red solution to a light pink suspension, indicating the successful generation of an MOF precursor. Then, a solvothermal reaction was used to further improve the crystallinity of the MOF. After sintering at a moderate temperature of 450 °C for 2 h with Ar protection, black Ni_x_CTO/C powder was collected, which proves that the organic groups in the precursor were transformed into carbonaceous materials in situ. To acquire carbon-free control samples, the precursor powder was heated under the same conditions but in an air atmosphere. It is noted that the as-prepared powder still exhibits a black color (Figure S1a in Supporting Information), owing to the incomplete removal of the organic groups. Consequently, various calcination conditions at elevated temperatures were systematically investigated (refer to Figure S1b–e). Notably, the color of the resulting powder transitioned to green at 600 °C, indicating the formation of pure Ni_x_CTO. Therefore, for the carbon-free control sample presented in this paper, a calcination condition of 600 °C for 5 h in air was selected.

**Figure 2:**
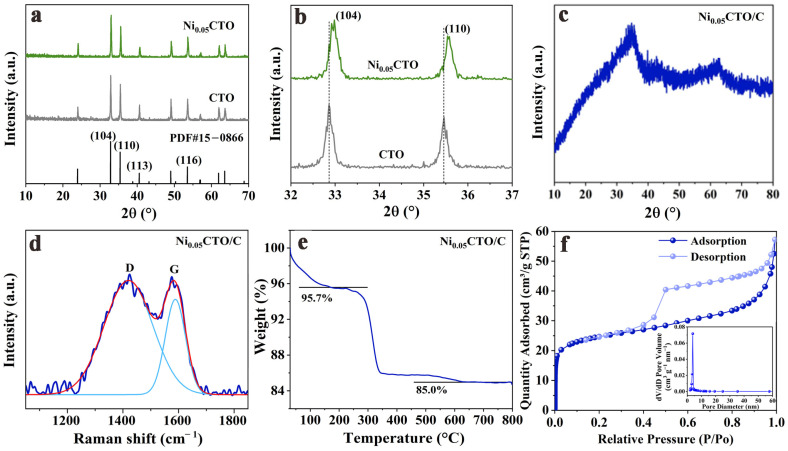
(**a**) XRD patterns and (**b**) the enlarged view of CTO and Ni_0.05_CTO. (**c**) XRD pattern of Ni_0.05_CTO/C. (**d**) Raman spectrum, (**e**) TG curve and (**f**) nitrogen adsorption–desorption isotherms and pore size distribution (inset) of Ni_0.05_CTO/C.

Powder X-ray diffraction (XRD) characterization was conducted to determine the composition and crystal structure of the as-prepared CTO samples. As illustrated in Figure 2a, both CTO and Ni_0.05_CTO exhibit distinct peaks that correspond well to the primary crystallographic planes of (012), (104), (110), (113), (024), (116), (214) and (300) for the CoTiO_3_ phase with an ilmenite structure (PDF card no.15-0866). Furthermore, no impurities are observed, confirming the high purity of the CTO samples. Figure 2b presents an enlarged view of the (104) and (110) peaks to validate the successful doping of nickel. Obviously, the diffraction peaks of Ni_0.05_CTO shift slightly towards higher angles compared to those of pristine CTO, which can be attributed to the substitution effect whereby Ni^2+^ replaces Co^2+^ ions. Given that Ni^2+^ has a smaller ionic radius than Co^2+^ (0.690Å versus 0.745 Å), the replacement results in a reduction in lattice spacing, which consequently leads to a rightward shift in peak positions for Ni_0.05_CTO. Moreover, calculations using the Scherrer formula based on the (104) plane reveal that Ni_0.05_CTO exhibits a decreased grain size of 57.4 nm compared to that of CTO (66.3 nm), thereby demonstrating that Ni doping facilitates grain refinement. Smaller grains are conducive to shortening the lithium-ion diffusion paths, which is beneficial for accelerating the electrochemical reaction kinetics. For Ni_0.05_CTO/C, shown in Figure 2c, broad peaks with low intensity are observed while carbon diffraction peaks are absent; this may be due to the existence of in situ-formed amorphous carbon along with small crystallite sizes resulting from carbon incorporation into the materials [14].

To verify the degree of graphitization and carbon content in Ni_0.05_CTO/C, Raman spectroscopy and thermogravimetric (TG) analysis were performed. The Raman spectrum shown in Figure 2d reveals distinct signals at around 1389.2 and 1575.2 cm^−1^, corresponding to the disorder carbon (D band) and graphitized carbon (G band), respectively [21]. The intensity ratio of the D band to G band (I_D_/I_G_) is measured at 2.87, indicating a low degree of graphitization and further confirming the presence of amorphous carbon, which aligns with the XRD results. The abundant disorder effects within the carbon structure are advantageous for increasing lithium storage sites, thereby enhancing electrochemical capacity. Furthermore, the TG curve depicted in Figure 2e illustrates two thermal weight loss steps occurring below 200 °C and between 250–500 °C. The initial step exhibits a minor weight loss of 4.3 wt%, attributed to moisture evaporation from the surface; conversely, the latter step arises from the oxidation of carbonaceous materials in the composite. Consequently, the carbon content in Ni_0.05_CTO/C is determined to be 10.7 wt%. In addition, nitrogen adsorption–desorption isotherms were analyzed along with their corresponding pore size distribution curves. As illustrated in Figure 2f, Ni_0.05_CTO/C displays typical IV-type curves, which are indicative of mesopores present within the carbon-coated composites [15]. Based on calculations using the BET model, its specific surface area is approximately 91.1 m^2^ g^−1^ with an average pore size of about 3.9 nm (inset). In contrast, CTO exhibits a specific surface area around 8.4 m^2^ g^−1^ alongside an average pore size of nearly 36 nm (Figure S2). The enhanced specific surface area observed for Ni_0.05_CTO/C can be ascribed to its smaller grain size, coupled with amorphous carbon’s presence within the composite material. The porous structure characterized by a high specific surface area facilitates complete electrolyte penetration into this material framework, thus enabling rapid lithium-ion transport during electrochemical reactions [17,22].

**Figure 3:**
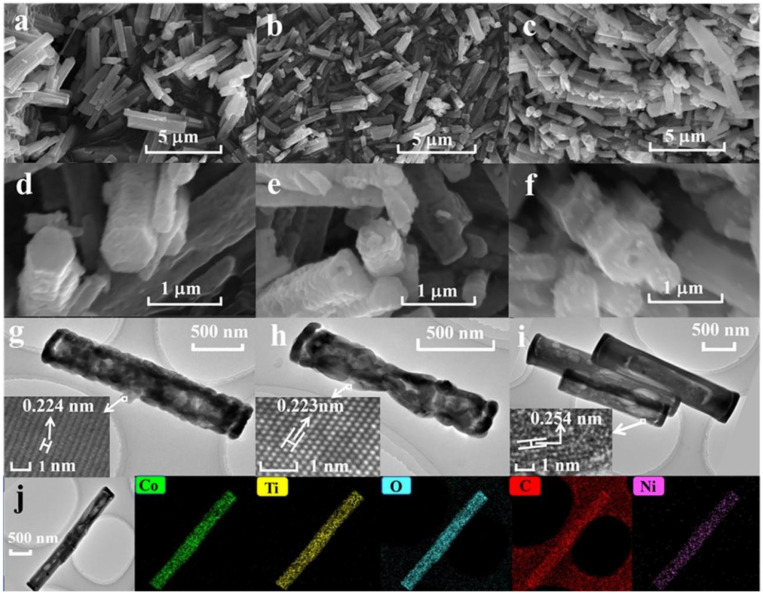
SEM images of (**a**,**d**) CTO, (**b**,**e**) Ni_0.05_CTO, and (**c**,**f**) Ni_0.05_CTO/C. TEM and HRTEM images of (**g**) CTO, (**h**) Ni_0.05_CTO, and (**i**) Ni_0.05_CTO/C (**j**). TEM image and corresponding elemental mappings of Ni_0.05_CTO/C.

The scanning electron microscopy (SEM) and transmission electron microscopy (TEM) images are presented in Figure 3. Figure 3a–f depict the SEM images of CTO, Ni_0.05_CTO, and Ni_0.05_CTO/C. It is evident that all three samples exhibit a one-dimensional morphology characterized by hexagonal micro-prisms, indicating that the microstructure remains intact after Ni doping. The length of an individual hexagonal micro-prism ranges from approximately 1 to 5 μm, with diameters between about 0.2 and 0.7 μm. High-magnification SEM images (Figure 3d–f) reveal that the carbon-free samples of CTO and Ni_0.05_CTO possess relatively rough surfaces compared to the Ni_0.05_CTO/C composite. Upon annealing in air, organic species are removed, leading to the formation of pores among interconnected nano-grains of CoTiO_3_; conversely, for Ni_0.05_CTO/C, these nano-grains are embedded within an in situ-formed carbon framework that was annealed in Ar atmosphere. The results obtained from TEM observations align closely with those observed via SEM techniques. More specifically, Figure 3g,h illustrate distinct voids present within the hexagonal micro-prisms of both CTO and Ni_0.05_CTO composed of primary nanoparticles. Furthermore, the high-resolution TEM (HRTEM) image shown as an inset in Figure 3g clearly demonstrates an interplanar spacing of 0.224 nm corresponding to the (113) plane of CoTiO_3_. Additionally, measurements depicted in Figure 3h,i show spacings of 0.223 nm and 0.254 nm attributed to the (113) and (110) planes of CoTiO_3_, respectively, which agrees with the results of XRD. Moreover, elemental mappings illustrated in Figure 3j indicate a uniform distribution of Co, Ti, O, C, and Ni elements throughout the Ni_0.05_CTO/C composite, further confirming the uniform doping of Ni element. The elemental mapping analyses for CTO and Ni_0.05_CTO can be found in Figure S3. It has been observed that only the elements of Ti, O, and Co are present in CTO, while carbon is absent in Ni_0.05_CTO. It should be noted that MOF-derived porous carbon not only serves as a matrix to mitigate the aggregation of active nanoparticle and volume changes during charge and discharge cycles, but also functions as a conductive network to facilitate the transport of Li^+^ ions and electrons [14,23]. Consequently, the unique one-dimensional structure of the Ni_0.05_CTO/C composite can significantly enhance both the efficiency and stability of the electrode material.

**Figure 4:**
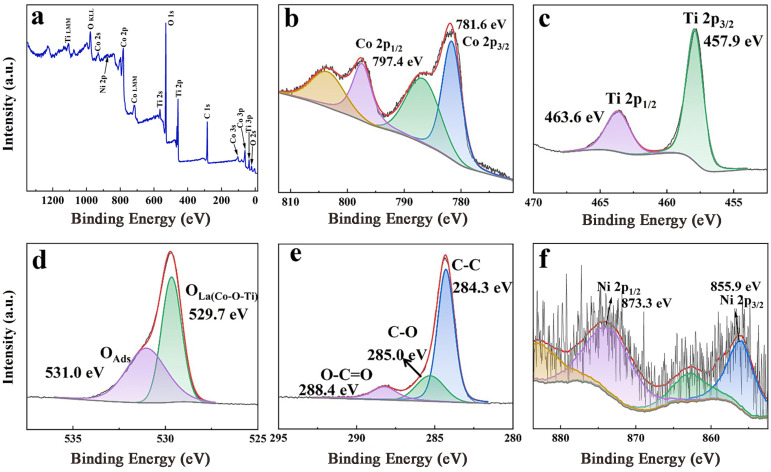
XPS spectra of Ni_0.05_CTO/C. (**a**) Full spectrum, (**b**) Co 2p, (**c**) Ti 2p, (**d**) O 1s, (**e**) C 1s, and (**f**) Ni 2p.

The chemical bonding and surface chemistry of Ni_0.05_CTO/C were investigated using X-ray photoelectron spectroscopy (XPS) analysis. Figure 4a presents the full spectrum, where clear peaks corresponding to Co, Ti, O, C, and Ni are observed, confirming the uniform elemental distribution within Ni_0.05_CTO/C. As illustrated in Figure 4b, the peak for Co can be deconvoluted into two main broad peaks located at approximately 797.4 and 781.6 eV, accompanied by their respective satellite peaks; these correspond to Co 2p_1/2_ and Co 2p_3/2_ states, respectively [22]. The binding energy difference between Co 2p_3/2_ and Co 2p_1/2_ is measured at 15.8 eV, thereby verifying the presence of bivalent cobalt [17]. For Ti 2p XPS (Figure 4c), two distinct peaks are observed at 463.6 and 457.9 eV, corresponding to Ti 2p_1/2_ and Ti 2p_3/2_, respectively. The energy difference of 5.7 eV between these two peaks further corroborates the presence of the oxidation state Ti^4+^ [17,24]. In the case of O 1s XPS (Figure 4d), two prominent peaks are centered at 529.7 and 531.0 eV. These main peaks can be attributed to characteristic lattice oxygen (O_La_) and surface chemisorbed oxygen (O_Ads_), which correspond to Co-O-Ti bonds and H-O interactions, respectively. Previous studies have indicated that the concentration of oxygen vacancies is directly proportional to the relative peak intensity ratio of O_Ads_ to O_La_ [25]. The intensity ratio of the O_Ads_ peak to the O_La_ peak in Ni_0.05_CTO/C (Figure 4d) is notably higher than that observed in CTO (Figure S4a), indicating an increased number of oxygen vacancies. Similarly, Ni_0.05_CTO also demonstrates a higher ratio (Figure S4b). This finding suggests that the incorporation of Ni into the CoTiO_3_ lattice may lead to an enhancement in oxygen vacancy concentration, which is beneficial for improving electronic conductivity, as previously reported [26]. Moreover, the C 1s spectrum can be deconvoluted into peaks centered at 288.4, 285.0, and 284.3 eV, as illustrated in Figure 4e. The peaks observed at 285.0 eV and 288.4 eV are indicative of the presence of C-O and O-C=O bonds, suggesting the existence of oxidized functional groups and oxides on the surface. The prominent peak at 284.3 eV signifies the presence of C-C bonds within the carbon material [27]. For Ni 2p XPS (Figure 4f), two prominent peaks are observed at 873.3 eV and 855.9 eV, corresponding to Ni 2p_1/2_ and Ni 2p_3/2_, respectively, due to spin-orbit splitting. Additionally, satellite peaks associated with these main peaks can be detected around 882.9 eV and 862.9 eV, which are attributed to the multiple splitting of the Ni 2p signal [28]. The atomic percentage of nickel in the Ni_0.05_CTO/C sample obtained from XPS is determined to be 0.67%. A summary of the atomic percentages for other elements is provided in Table S1. Furthermore, it is noted that the actual Ni doping content closely aligns with the theoretical value when x = 0.05 in the starting materials.

**Figure 5:**
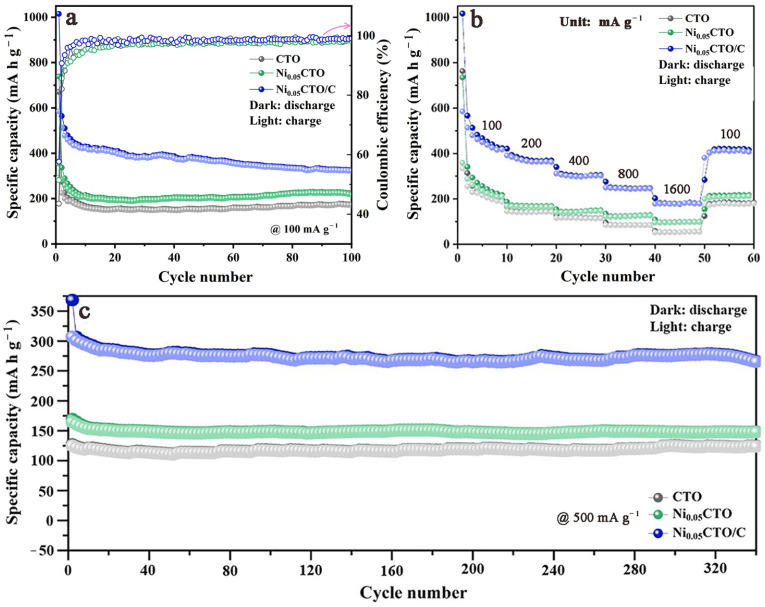
(**a**) Cycling performance and Coulombic efficiency at 100 mA g^−1^, (**b**) rate capability at varied current densities, and (**c**) long-term cycling performance at 500 mA g^−1^ of CTO, Ni_0.05_CTO, and Ni_0.05_CTO/C.

As fast charging performance is a critical factor for the practical application of materials, the electrochemical performance of pristine CTO and single modified Ni_x_CTO at various current densities was initially evaluated to investigate the effects of Ni doping. As illustrated in Figure S5, the Ni-doped CTO samples (with x = 0.02, 0.05, 0.08) demonstrate enhanced rate capability compared to the pristine sample. Notably, Ni_0.05_CTO with a moderate level of Ni doping exhibits superior performance, particularly at high current rates of 800 and 1600 mA g^−1^. Consequently, in subsequent discussions, the Ni_0.05_CTO sample will be utilized. Figure 5a compares the cycling performance of pristine CTO, single modified Ni_0.05_CTO, and dually modified Ni_0.05_CTO/C at 100 mA g^−1^. The initial discharge/charge capacities for Ni_0.05_CTO/C are 1014.7/584.8 mA h g^−1^ with an initial Coulombic efficiency (ICE) of 57.6%. As for Ni_0.05_CTO, the initial discharge/charge capacities are 738.8/353.9 mA h g^−1^ with an ICE of 47.9%, both values being inferior to those observed for Ni_0.05_CTO/C. Furthermore, pristine CTO displays the lowest capacities at only 670.5/292.0 mA h g^−1^ with an ICE of 43.5%. Throughout repeated lithiation/de-lithiation cycles, all three samples exhibit capacity fading during their initial cycles; however, it is noteworthy that carbon involvement leads to more rapid capacity decay in Ni_0.05_CTO/C. This phenomenon may be attributed to significant decomposition of the electrolyte occurring on the carbon surface, which forms a solid electrolyte interface (SEI) film resulting in irreversible capacity loss. After completing one hundred cycles, the Ni_0.05_CTO/C exhibits a high reversible capacity of 322.0 mA h g^−1^, whereas Ni_0.05_CTO and CTO demonstrate significantly lower capacities of 228.5 and 174.0 mA h g^−1^, respectively.

The rate capabilities of the three samples are compared in Figure 5b. The average discharge capacity of Ni_0.05_CTO/C at each current rate is measured to be 421.3, 367.3, 302.4, 245.2, and 182.0 mA h g^−1^, sequentially. When the current density is returned to 100 mA g^−1^, the capacity recovers to 420.9 mA h g^−1^, which is nearly identical to that observed during previous cycling at this same current rate, thereby confirming its excellent cycling reversibility. All capacity values are summarized in Table 1; notably, the rate capability of Ni_0.05_CTO/C significantly surpasses that of both Ni_0.05_CTO and CTO samples. Furthermore, it has been observed that Ni_0.05_CTO demonstrates a higher capacity than CTO, even at a lower rate; this enhancement can be attributed to the smaller grain size induced by Ni doping, which facilitates lithium-ion diffusion and electrochemical reactions. The long-term cycling performance at a high current density of 500 mA g^−1^ is further illustrated in Figure 5c. A significant reversible capacity of 273.3 mA h g^−1^ for Ni_0.05_CTO/C remains after undergoing 340 cycles—this value markedly exceeds those recorded for CTO (121.0 mA h g^−1^) and Ni_0.05_CTO (152.0 mA h g^−1^). Based on these results, it is evident that the dually modified Ni_0.05_CTO/C exhibits the best performance among all the samples; such enhanced electrochemical properties can be ascribed to the synergistic effects arising from Ni doping combined with in situ carbon involvement.

**Figure 6:**
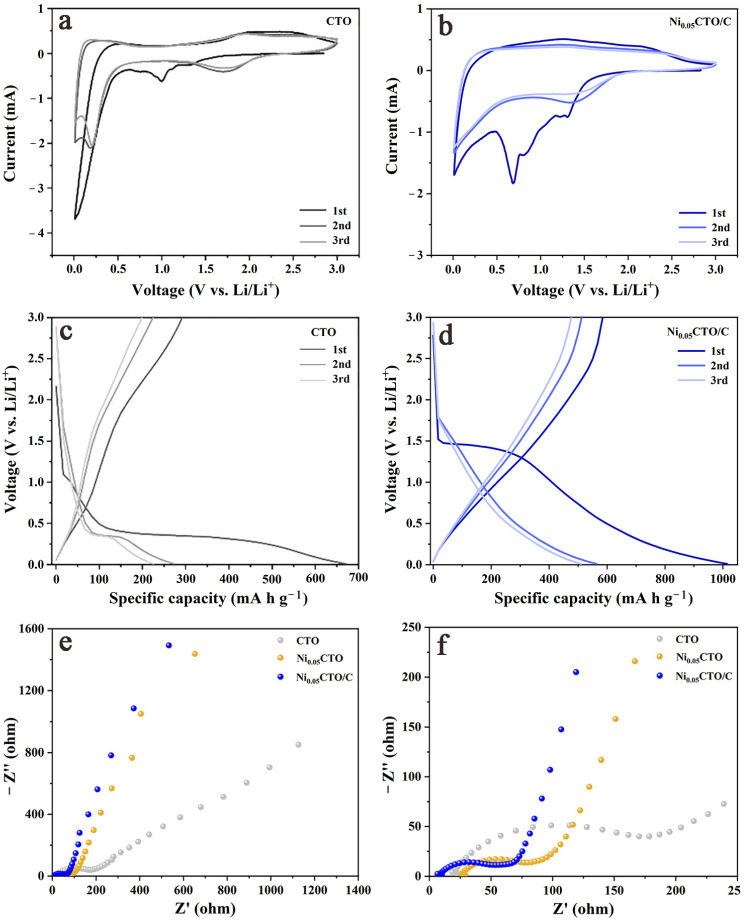
CV curves of (**a**) CTO and (**b**) Ni_0.05_CTO/C. Charge/discharge profiles of (**c**) CTO and (**d**) Ni_0.05_CTO/C. (**e**) EIS spectra and (**f**) the enlarged view for CTO, Ni_0.05_CTO, and Ni_0.05_CTO/C electrodes measured at OCV.

The electrochemical reactions occurring during the charge/discharge process of CTO and Ni_0.05_CTO/C were investigated using cyclic voltammetry (CV) curves. As illustrated in Figure 6a,b, both CTO and Ni_0.05_CTO/C exhibit four reduction peaks at approximately 1.3, 1.0, 0.75, and 0.2 V during the first cathodic scan. The peak observed at 1.3 V can be attributed to the Ti^4+^ to Ti^3+^ transition upon Li^+^ intercalation into the CoTiO_3_ crystal lattice [29]. The two irreversible peaks located at 1.0 and 0.75 V are likely due to the formation of an SEI film [30,31], which accounts for the irreversible capacity loss that disappears in subsequent cycles. The peak at 0.2 V corresponds to the reduction of Co^2+^ to Co^0^ [30,32]. During the first anodic scan, two oxidation peaks appear around 2.0 and 2.3 V; these are associated with reversible Li^+^ extraction from the TiO_2_ crystal lattice and transformation of Co^0^ to Co^2+^, respectively [33]. From the second cycle onward, the CV curves show a significant overlap with a pair of broad redox peaks near 1.7/2.2 V, indicating good electrochemical reversibility following SEI formation and structural rearrangement. The discharge/charge profiles for the initial three cycles (Figure 6c,d) further corroborate these findings by demonstrating similar characteristics: voltage plateaus, along with capacity loss, are evident during the first cycle, consistent with observations from the CV curves.

To gain a deeper understanding of the differences in lithium storage performance, electrochemical impedance spectroscopy (EIS) was employed to elucidate the kinetic processes involved. Figure 6e illustrates that the Nyquist plots for CTO, Ni_0.05_CTO, and Ni_0.05_CTO/C measured at open-circuit voltage (OCV) exhibit remarkable similarity; they consist of a semicircle in the high-to-medium frequency region and an inclined line in the low-frequency region. The semicircle is associated with the charge transfer resistance (R_ct_), which depends on the radius of the semicircle, at the electrode/electrolyte interfaces, while the inclined line represents Li^+^ diffusion within the active material [29,34]. As shown in Figure 6f, there is a clear trend regarding R_ct_ values from smallest to largest among the three samples: Ni_0.05_CTO/C < Ni_0.05_CTO < CTO. The lowest R_ct_ observed for Ni_0.05_CTO/C can be attributed to both Ni doping and carbon incorporation into the material, facilitating faster electron transport. Furthermore, Figure S6 presents a relationship between Z’ and ω^−1/2^ in the low-frequency region; here, a smaller slope indicates enhanced Li^+^ diffusion within the electrode material. After performing linear fitting on these curves, we obtained slopes of 268.8, 147.5, and 88.7 for CTO, Ni_0.05_CTO, and Ni_0.05_CTO/C, respectively. It is evident that after Ni doping, there is an improvement in Li^+^ diffusion kinetics across all samples; notably, Ni_0.05_CTO/C exhibits the smallest slope, which is indicative of a superior Li^+^ diffusion rate in the electrode compared to others tested herein. In conclusion, it can be inferred that Ni_0.05_CTO/C demonstrates significantly enhanced electrochemical performance as a result of ameliorated kinetic processes.

**Figure 7:**
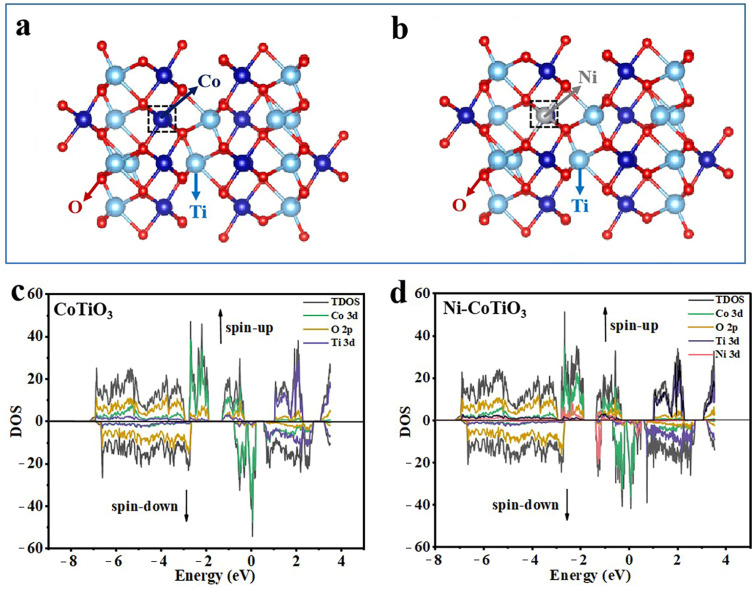
The structure models of (**a**) CoTiO_3_ and (**b**) Ni-doped CoTiO_3_. Calculated density of states (DOS) of (**c**) CoTiO_3_ and (**d**) Ni-doped CoTiO_3_.

Density functional theory (DFT) calculations were conducted to investigate the doping configuration of Ni-doped CoTiO_3_ and to examine the impact of Ni doping on the modulation of the electronic structure of CoTiO_3_. In these calculations, periodically repeated single crystal models of CoTiO_3_ were employed, considering two potential cases for Ni doping, as illustrated in Figure S7 for the optimized results. The calculated energies indicate that when Ni atoms substitute for Co atoms, the electronic energy (E_0_) is −244.69 eV; conversely, when Ni atoms replace Ti atoms, E_0_ is −237.12 eV. This finding suggests a greater likelihood for Ni atoms to replace Co rather than Ti. The DFT calculation results are consistent with the aforementioned XRD analysis and further substantiate that the doping configuration involves Ni substituting for Co in doped CoTiO_3_ samples. Additionally, the computed total and partial density of states (TDOS/PDOS) for both CoTiO_3_ and its Ni-doped variant—based on their respective crystal models (Figure 7)—indicate a slight enhancement in conductivity following Ni doping. This improvement arises from new local energy levels (impurity energy levels), attributed to minor hybridization effects involving Ni 3d orbitals appearing within the forbidden band below the Fermi level. Consequently, it can be concluded that Ni doping enhances the electronic conductivity of CoTiO_3_, thereby elucidating its improved electrochemical performance.

## 3. Materials and Methods

### 3.1. Materials Preparation

In a typical synthesis route for Ni_x_Co_1−x_TiO_3_ (x = 0.02, 0.05, 0.08) without carbon coating, stoichiometric amounts of urea, cobalt acetate tetrahydrate, nickel acetate tetrahydrate, and tetrabutyl titanate in a molar ratio of 3:1 − x:x:1 were initially dissolved in 60 mL of ethylene glycol to form a transparent red solution. After rigorous stirring for 2 h, the solution transformed into a light pink micellar suspension. Then the pink solution was transferred into a stainless steel autoclave and maintained at 120 °C for 6 h. Upon cooling to room temperature, the pink precursor was collected after thorough centrifugation and washing with ethanol three times. Subsequently, the dried precursor powder was sintered in a furnace at 600 °C for 5 h in air with a heating rate of 5 °C min^−1^. Ultimately, pure Ni-doped CTO sample was obtained and labeled as Ni_x_CTO.

For the synthesis of the CoTiO_3_ sample without Ni doping (named as CTO), the procedure is identical to that used for Ni_x_CTO, except that no nickel acetate tetrahydrate was added in the starting materials.

For the synthesis of dually modified CTO samples (Ni_x_CTO/C, where x = 0.02, 0.05, 0.08), the procedure closely resembles that used for Ni_x_CTO, except that the obtained precursor was sintered at 450 °C for a duration of 2 h under the protection of Ar atmosphere. For better comparison, the dually modified CTO sample Ni_0.05_CTO/C was selected for investigation in this study. The quantities of reagents utilized for each sample and the corresponding calcination conditions are summarized in Table S2.

### 3.2. Materials Characterization

Ultra-high-resolution field emission scanning electron microscopy (SEM, Nova NanoSEM 450, FEI, Brno, Czech Republic) and high-resolution transmission electron microscopy (HRTEM, TalosF200X G2, Thermo Fisher Scientific, Hillsboro, OR, USA) were used for the morphological characterization of the as-prepared products. An X-ray diffractometer (Utima IV, Rigaku, Tokyo, Japan) was used to measure the X-ray diffraction (XRD) patterns of the samples under the condition of Cu Kα radiation. The automatic adsorption device (ASAP 2460, Micromeritics, Norcross, GA, USA) was used to obtain nitrogen adsorption/desorption isotherms at 77 K, and the surface area was calculated based on the Brunauer–Emmett–Teller (BET) equation. Raman spectra were recorded by UK Renishaw equipped with a 532 nm laser at room temperature. Thermogravimetric analysis (TG, STA 449F5, NETZSCH, Gerätebau GmbH, Barsbüttel, Germany) was used to analyze the carbon content in the sample. X-ray photoelectron spectroscopy (XPS, ESCALAB XI+, Thermo Fisher Scientific, Waltham, MA, USA) was used to determine the elemental chemical bonding state.

### 3.3. Electrochemical Tests

CR2025-type coin cells were assembled to study the electrochemical performance of the samples. To make the working electrodes, uniform slurry was firstly prepared by mixing the active materials, acetylene black, and polyvinylidene fluoride with a mass ratio of 8:1:1 in N-methylpyrrolidinone solvent and then coating it on the copper foil. After drying at 80 °C overnight in vacuum, the copper foil was cut into small disks with a diameter of 14 mm. The mass loading of active materials on each electrode was about 3 mg cm^−2^. The cell assembly procedure was carried out in an argon-filled glove box, and 1 M LiPF_6_ dissolved in ethyl carbonate and diethyl carbonate (1:1 in volume) with 5% fluoroethylene carbonate additive was adopted as electrolyte. Celgard 2325 microporous polypropylene film was employed as the separator, and lithium foil was used as both the reference and counter electrode. The galvanostatic charge/discharge test was performed on a Neware battery test system within a voltage range of 0.01 to 3.0 V (vs. Li/Li^+^). Cyclic voltammetry (CV) was performed on an IviumStat electrochemistry workstation at a scanning rate of 0.3 mV s^−1^ within the same voltage range. An electrochemical impedance spectroscopy (EIS) test for the cells was carried out at OCV in a frequency range of 0.01 Hz to 100 kHz with the disturbance amplitude of 5 mV.

### 3.4. Theoretical Calculations

The spin-polarized density functional theory (DFT) method was employed for all calculations using the Perdew–Burke–Ernzerhof functional as implemented in the Vienna Ab initio Simulation Package (VASP, version 5.4.4) [35]. The exchange-correlation functional under the generalized gradient approximation, with projector augmented wave pseudo-potentials and the Perdew–Burke–Ernzerhof functional, were adopted to describe the electron–electron interaction [36]. The plane-wave cutoff was set to 450 eV. The Brillouin zone was sampled using a 13 × 13 × 5 k-point grid for the calculations of density of states (DOS) and a 1 × 5 × 2 k-point sampling for structural optimizations. A force tolerance of 0.02 eV Å^−1^ and energy tolerance of 5.0 × 10^−6^ eV per atom were considered. Data processing was performed using the VASPKIT package (version 1.4.1).

## 4. Conclusions

In conclusion, MOF-derived Ni-doped CoTiO_3_/amorphous carbon hexagonal micro-prisms were synthesized using a simple solvothermal method. The substitutional doping of Ni for Co does not destroy the unique 1D structure of the as-prepared CoTiO_3_. Enhanced electronic conductivity and improved lithium-ion diffusion kinetics resulting from Ni doping contribute to the superior electrochemical performance of doped CoTiO_3_ micro-prisms, even in the absence of carbon coating, compared to their pristine counterparts. More importantly, when coupled with in situ-formed carbon, the dually modified composites exhibit significantly enhanced lithium storage capacity along with exceptional rate capability and cycling stability due to synergistic effects. The simple dual-modification strategy proposed in this study can be extended to modify and prepare other electrode materials for advanced rechargeable batteries.

## Figures and Tables

**Table 1:** Discharge capacities (mA h g^−1^) after 100 cycles at 100 mA g^−1^ and the average discharge capacities under different current densities (mA g^−1^).

Sample	Capacity (100th Cycle)	Capacities at Varied Current Densities					
		100	200	400	800	1600	100
CTO	174.0	196.0	143.6	117.0	84.4	54.2	185.0
Ni_0.05_CTO	228.5	218.2	168.1	144.8	124.9	98.0	217.1
Ni_0.05_CTO/C	322.0	421.3	367.3	302.4	245.2	182.0	420.9

## Data Availability

The data that support the findings of this study are available from the corresponding author upon reasonable request.

## Supplementary Materials

The following supporting information can be downloaded at: https://www.mdpi.com/article/10.3390/molecules30010034/s1, Figure S1: Photo images of the products obtained under different conditions in air; Figure S2: (a) Nitrogen adsorption–desorption isotherms and (b) pore size distribution of CTO; Figure S3: HAADF-STEM images and corresponding elemental mappings of CTO and Ni_0.05_CTO; Figure S4: XPS spectra of O 1s of (a) CTO and (b) Ni_0.05_CTO; Figure S5: Rate capability (charge capacity) comparison of CTO, Ni_0.02_CTO, Ni_0.05_CTO, and Ni_0.08_CTO; Figure S6: Relationship between Z’ and ω^−1/2^ at low frequency of CTO, Ni_0.05_CTO, and Ni_0.05_CTO/C; Figure S7: The structure models for the replacement of (a) Ti and (b) Co sites by Ni atoms; Table S1: The atomic percentages of the elements present in Ni_0.05_CTO/C obtained from XPS; Table S2: The amounts of reagents used for each sample and the calcination conditions.

## References

[1] Kim T., Song W., Son D.-Y., Ono L.K., Qi Y. (2019). Lithium-ion batteries: Outlook on present, future, and hybridized technologies. J. Mater. Chem. A.

[2] Wulandari T., Fawcett D., Majumder S.B., Poinern G.E.J. (2023). Lithium-based batteries, history, current status, challenges, and future perspectives. Battery Energy.

[3] Xu J., Cai X., Cai S., Shao Y., Hu C., Lu S., Ding S. (2023). High-Energy Lithium-Ion Batteries: Recent Progress and a Promising Future in Applications. Energy Environ. Mater..

[4] Cheng H., Shapter J.G., Li Y., Gao G. (2021). Recent progress of advanced anode materials of lithium-ion batteries. J. Energy Chem..

[5] Liu S., Gu B., Chen Z., Zhan R., Wang X., Feng R., Sun Y. (2024). Suppressing dendritic metallic Li formation on graphite anode under battery fast charging. J. Energy Chem..

[6] Fang S., Bresser D., Passerini S. (2020). Transition Metal Oxide Anodes for Electrochemical Energy Storage in Lithium- and Sodium-Ion Batteries. Adv. Energy Mater..

[7] Shi H., Shi C., Jia Z., Zhang L., Wang H., Chen J. (2022). Titanium dioxide-based anode materials for lithium-ion batteries: Structure and synthesis. RSC Adv..

[8] Muchuweni E., Mombeshora E.T., Muiva C.M., Sathiaraj T.S. (2023). Lithium-ion batteries: Recent progress in improving the cycling and rate performances of transition metal oxide anodes by incorporating graphene-based materials. J. Energy Storage.

[9] Zhu J., Ding Y., Ma Z., Tang W., Chen X., Lu Y. (2022). Recent Progress on Nanostructured Transition Metal Oxides As Anode Materials for Lithium-Ion Batteries. J. Electron. Mater..

[10] Zhang J., Yu A. (2015). Nanostructured transition metal oxides as advanced anodes for lithium-ion batteries. Sci. Bull..

[11] Li N., Zheng P., Wang R., Zhao X. (2024). Co-doped MnO_2_ nanorods with oxygen vacancies as anode for Li-ion battery. J. Mater. Sci. Mater. Electron..

[12] Bai X., Li T., Qi Y.-X., Wang Y.-X., Yin L.-W., Li H., Lun N., Bai Y.-J. (2016). One-step fabricating nitrogen-doped TiO_2_ nanoparticles coated with carbon to achieve excellent high-rate lithium storage performance. Electrochim. Acta.

[13] Yu G., Zhang Q., Jing J., Wang X., Li Y., Bai X., Li T. (2023). Bulk Modification of Porous TiNb_2_O_7_ Microsphere to Achieve Superior Lithium-Storage Properties at Low Temperature. Small.

[14] Ouyang B., Chen T., Qin R., Liu P., Fan X., Wang J., Liu W., Liu K. (2021). Bimetal–organic-framework derived CoTiO_3_/C hexagonal micro-prisms as high-performance anode materials for metal ion batteries. Mater. Chem. Front..

[15] Huang Z.-D., Zhang T.-T., Lu H., Masese T., Yamamoto K., Liu R.-Q., Lin X.-J., Feng X.-M., Liu X.-M., Wang D. (2018). Grain-boundary-rich mesoporous NiTiO_3_ micro-prism as high tap-density, super rate and long life anode for sodium and lithium ion batteries. Energy Storage Mater..

[16] Ghaemifar S., Rahimi-Nasrabadi M., Pourmasud S., Eghbali-Arani M., Behpour M., Sobhani-Nasab A. (2020). Preparation and characterization of MnTiO_3_, FeTiO_3_, and CoTiO_3_ nanoparticles and investigation various applications: A review. J. Mater. Sci. Mater. Electron..

[17] Liu S.-Y., Fan C.-Y., Wang H.-C., Zhang J.-P., Wu X.-L. (2017). Electrochemical In Situ Formation of a Stable Ti-Based Skeleton for Improved Li-Storage Properties: A Case Study of Porous CoTiO_3_ Nanofibers. Chem.-Eur. J..

[18] Liu W., Yin R., Xu X., Zhang L., Shi W., Cao X. (2019). Structural Engineering of Low-Dimensional Metal–Organic Frameworks: Synthesis, Properties, and Applications. Adv. Sci..

[19] Radwan A., Jin H., He D., Mu S. (2021). Design Engineering, Synthesis Protocols, and Energy Applications of MOF-Derived Electrocatalysts. Nano-Micro Lett..

[20] Huang Z.-D., Zhang T.-T., Lu H., Yang J., Bai L., Chen Y., Yang X.-S., Liu R.-Q., Lin X.-J., Li Y. (2018). Bimetal-organic-framework derived CoTiO3 mesoporous micro-prisms anode for superior stable power sodium ion batteries. Sci. China Mater..

[21] Li T., Wei C., Wu Y.-M., Han F., Qi Y.-X., Zhu H.-L., Lun N., Bai Y.J. (2015). Simple preparation of carbon nanofibers with graphene layers perpendicular to the length direction and the excellent li-ion storage performance. ACS Appl. Mater. Interfaces.

[22] Guo D., Xu Y., Xu J., Guo K., Wu N., Cao A., Liu G., Liu X. (2024). Synergistic Engineering of CoO/MnO Heterostructures Integrated with Nitrogen-Doped Carbon Nanofibers for Lithium-Ion Batteries. Molecules.

[23] Li J., Wang D., Zhou J., Hou L., Gao F. (2019). MOF-derived in situ synthesized carbon-coated ilmenite cobalt titanate nanocrystalline, high-stability lithium-ion batteries. J. Alloys Compd..

[24] Li Y., Fang Z., Feng L., Liu F., Shi Y., Li J., Zhao C. (2024). Study of Mesoporous Zr-TiO_2_ Catalyst with Rich Oxygen Vacancies for N-Methylmorpholine Oxidation to N-Methylmorpholine-N-oxide. Molecules.

[25] Tan T., Du Y., Cao A., Sun Y., Zha G., Lei H., Zheng X. (2018). The resistive switching characteristics of Ni-doped HfO_x_ film and its application as a synapse. J. Alloys Compd..

[26] Yu G., Huang J., Bai X., Li T., Song S., Zhou Y., Wu N., Yao S., Lu X., Wu W. (2024). Engineering of Cerium Modified TiNb_2_O_7_ Nanoparticles for Low-Temperature Lithium-Ion Battery. Small.

[27] Rehman W.u., Huang H., Yousaf M.Z., Aslam F., Wang X., Ghani A. (2023). Porous Carbon with Alumina Coating Nanolayer Derived from Biomass and the Enhanced Electrochemical Performance as Stable Anode Materials. Molecules.

[28] Abdel-wahab M.S., Jilani A., Yahia I.S., Al-Ghamdi A.A. (2016). Enhanced the photocatalytic activity of Ni-doped ZnO thin films: Morphological, optical and XPS analysis. Superlattices Microstruct..

[29] Li T., Yu G., Song M., Zhang Q., Li Y., Bai X. (2023). Facile Synthesis of Nb-Doped CoTiO_3_ Hexagonal Microprisms as Promising Anode Materials for Lithium-Ion Batteries. Inorganics.

[30] Wang X., Cheng W., Hu J., Su Y., Kong X., Uemura S., Kusunose T., Feng Q. (2021). Lithium Ion Battery Anode of Mesocrystalline CoTiO_3_/TiO_2_ Nanocomposite with Extremely Enhanced Capacity. ACS Appl. Energy Mater..

[31] Sun Y., Hu X., Luo W., Xia F., Huang Y. (2013). Reconstruction of Conformal Nanoscale MnO on Graphene as a High-Capacity and Long-Life Anode Material for Lithium Ion Batteries. Adv. Funct. Mater..

[32] Wang F., Zhuo H.-Y., Han X., Chen W.-M., Sun D. (2017). Foam-like CoO@N,S-codoped carbon composites derived from a well-designed N,S-rich Co-MOF for lithium-ion batteries. J. Mater. Chem. A.

[33] Sun M., Sheng X., Li S., Cui Z., Li T., Zhang Q., Xie F., Wang Y. (2022). Construction of porous CoTiO_3_ microrods with enhanced performance as lithium-ion battery anode. J. Alloys Compd..

[34] Tang Y., Wu L., Xiao L., Wen D., Guo Q., Liang W. (2018). Porous CoTiO_3_ microbars as super rate and long life anodes for sodium ion batteries. Ceram. Int..

[35] Kresse G., Furthmüller J. (1996). Efficient iterative schemes for ab initio total-energy calculations using a plane-wave basis set. Phys. Rev. B.

[36] Segall M.D., Shah R., Pickard C.J., Payne M.C. (1996). Population analysis of plane-wave electronic structure calculations of bulk materials. Phys. Rev. B.

