# Supplementary material for: Engineering of Metal–Organic Framework-Derived CoTiO3 Micro-Prisms for Lithium-Ion Batteries
Source: Molecules. 2024 Dec 26;30(1):34. doi: 10.3390/molecules30010034 (PMC11721643; doi:10.3390/molecules30010034)
Supplement: Supplementary file 1 [file molecules-30-00034-s001.zip › molecules-3217513-supplementary.pdf]

Supporting Information

Engineering of Metal–Organic Framework-Derived $CoTiO_3$ Micro-Prisms for Lithium-Ion Batteries

Tao Li [1,*], Minghui Song [2], Qi Zhang [3], Yifan Li [3], Gengchen Yu [2] and Xue Bai [1,2]

[1] Department of Materials Engineering, Xuzhou College of Industrial Technology, Xuzhou 221140, China
[2] School of Materials Science and Engineering, Shandong University of Science and Technology, Qingdao 266590, China
[3] School of Environmental and Municipal Engineering, Qingdao University of Technology, Qingdao 266033, China
* Correspondence: lit@mail.xzcit.cn

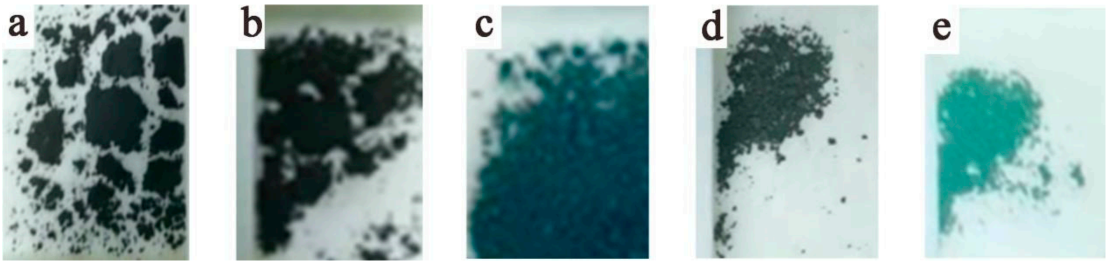

Figure S1. Photo images of the products obtained under different conditions in air: (a) 450 °C, 2 h; (b) 450 °C, 5 h; (c) 500 °C, 5 h; (d) 550 °C, 5 h; (e) 600 °C, 5 h.

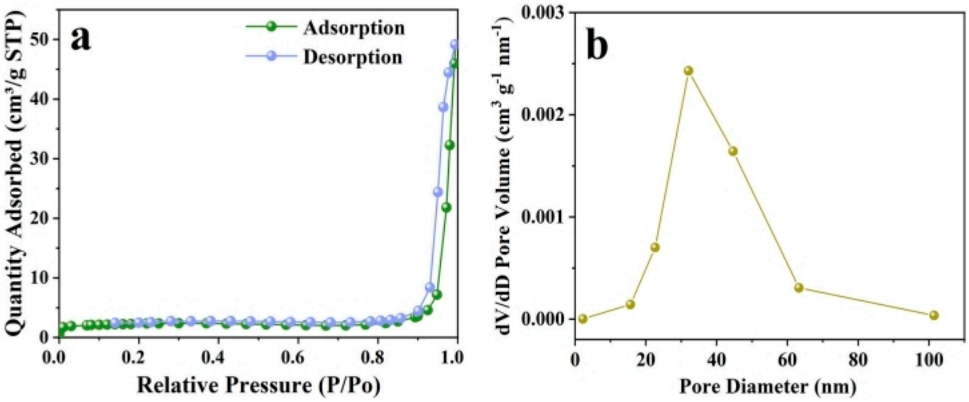

Figure S2. (a) Nitrogen adsorption–desorption isotherms and (b) pore size distribution of CTO.

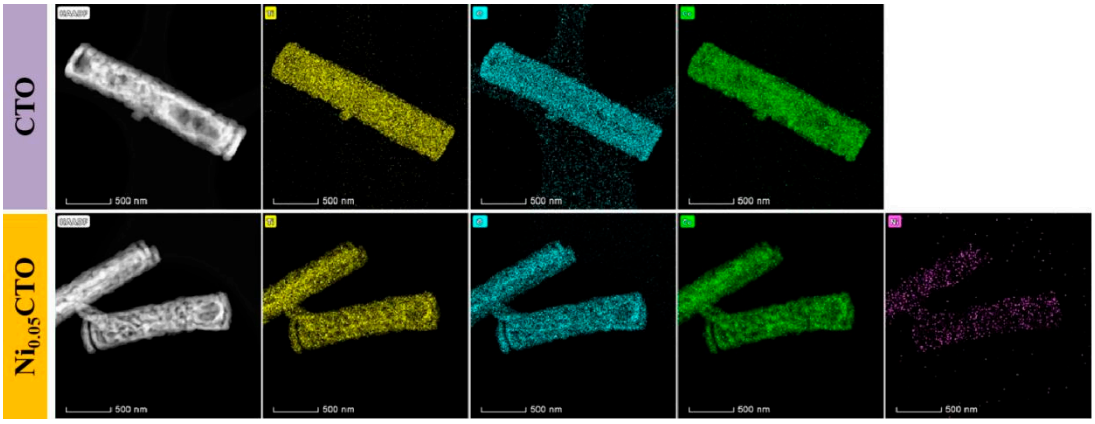

Figure S3. HAADF-STEM images and corresponding elemental mappings of CTO and $Ni_{0.05}CTO$.

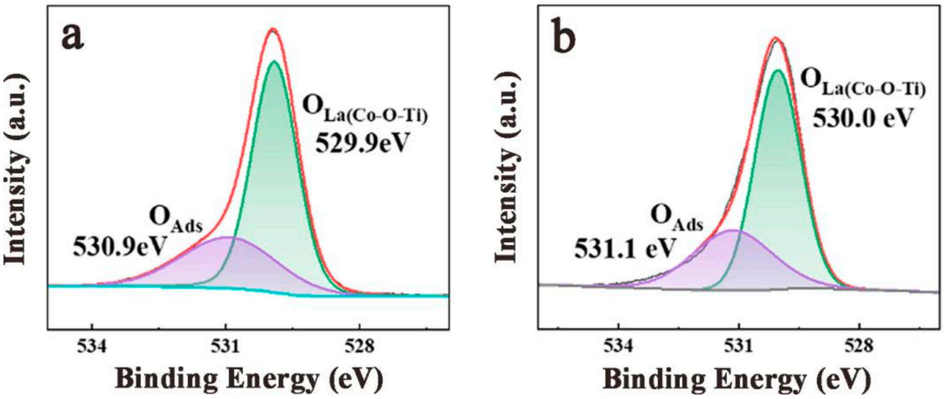

Figure S4. XPS spectra of O 1s of (a) CTO and (b) $Ni_{0.05}CTO$.

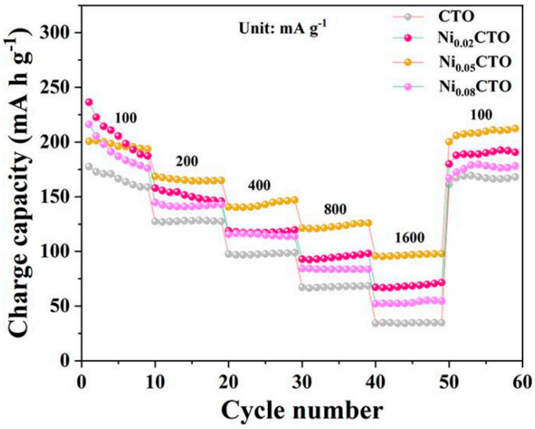

Figure S5. Rate capability (charge capacity) comparison of CTO, $Ni_{0.02}CTO$, $Ni_{0.05}CTO$, and $Ni_{0.08}CTO$.

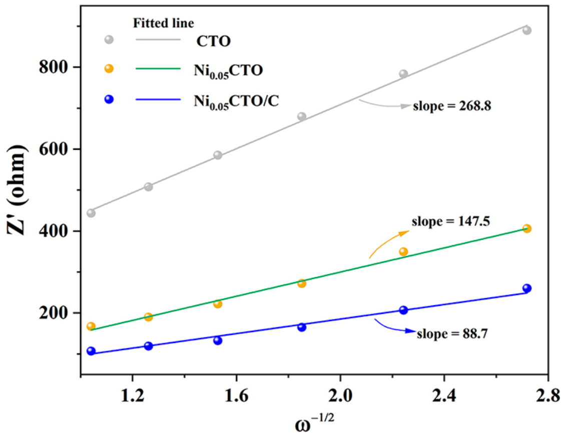

Figure S6. Relationship between Z′ and $\omega^{-1/2}$ at low frequency of CTO, $Ni_{0.05}$CTO, and $Ni_{0.05}$CTO/C.

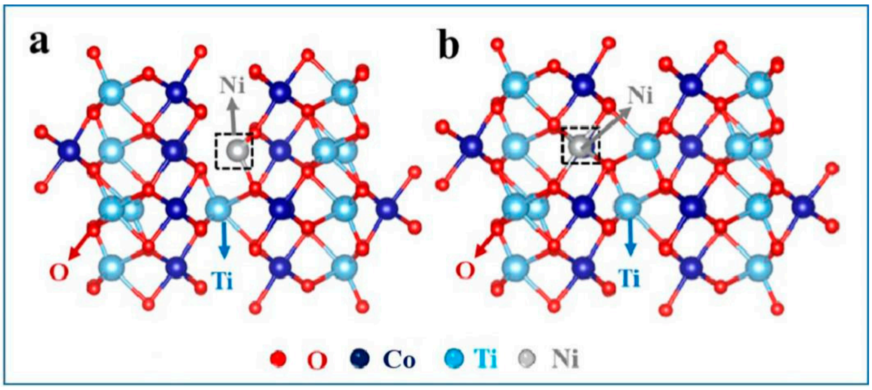

Figure S7. The structure models for the replacement of (a) Ti and (b) Co sites by Ni atoms.

Table S1. The atomic percentages of the elements present in $Ni_{0.05}$CTO/C obtained from XPS.

Element	Percentage (at%)
Ti2p	10.94
C1s	37.23
Co2p	10.16
Ni2p	0.67
O1s	41

Table S2. The amounts of reagents used for each sample and the calcination conditions.

	Reagents and amounts				
**Sample**	Nickel acetate tetrahydrate	Cobalt acetate tetrahydrate	Tetrabutyl titanate	Urea	**Calcination conditions**
CTO	--	2 mmol	2 mmol	6 mmol	600 °C- 5 h, air
$Ni_{0.02}CTO$	0.04 mmol	1.96 mmol	2 mmol	6 mmol	600 °C- 5 h, air
$Ni_{0.05}CTO$	0.1 mmol	1.9 mmol	2 mmol	6 mmol	600 °C- 5 h, air
$Ni_{0.08}CTO$	0.16 mmol	1.84 mmol	2 mmol	6 mmol	600 °C- 5 h, air
$Ni_{0.05}CTO/C$	0.1 mmol	1.9 mmol	2 mmol	6 mmol	450 °C- 2 h, Ar
